# Associations between intraoperative ventilator settings during one-lung ventilation and postoperative pulmonary complications: a prospective observational study

**DOI:** 10.1186/s12871-018-0476-x

**Published:** 2018-01-25

**Authors:** Shuji Okahara, Kazuyoshi Shimizu, Satoshi Suzuki, Kenzo Ishii, Hiroshi Morimatsu

**Affiliations:** 1 Department of Anesthesiology and Resuscitology, Okayama University Graduate School of Medicine, Dentistry and Pharmaceutical Sciences, 2-5-1 Shikata-cho, Kita-ku, Okayama, 700-8558 Japan; 2 Department of Anesthesiology and Oncological Pain Medicine, Fukuyama City Hospital, 5-23-1, Zaocho, Fukuyama-shi, Hiroshima 721-8511 Japan

**Keywords:** One-lung ventilation, Current practice of intraoperative ventilation., Inspired oxygen fraction., Postoperative pulmonary complications., Lung protective ventilation

## Abstract

**Background:**

The interest in perioperative lung protective ventilation has been increasing. However, optimal management during one-lung ventilation (OLV) remains undetermined, which not only includes tidal volume (V_T_) and positive end-expiratory pressure (PEEP) but also inspired oxygen fraction (F_I_O_2_). We aimed to investigate current practice of intraoperative ventilation during OLV, and analyze whether the intraoperative ventilator settings are associated with postoperative pulmonary complications (PPCs) after thoracic surgery.

**Methods:**

We performed a prospective observational two-center study in Japan. Patients scheduled for thoracic surgery with OLV from April to October 2014 were eligible. We recorded ventilator settings (F_I_O_2_, V_T_, driving pressure (ΔP), and PEEP) and calculated the time-weighted average (TWA) of ventilator settings for the first 2 h of OLV. PPCs occurring within 7 days of thoracotomy were investigated. Associations between ventilator settings and the incidence of PPCs were examined by multivariate logistic regression.

**Results:**

We analyzed perioperative information, including preoperative characteristics, ventilator settings, and details of surgery and anesthesia in 197 patients. Pressure control ventilation was utilized in most cases (92%). As an initial setting for OLV, an F_I_O_2_ of 1.0 was selected for more than 60% of all patients. Throughout OLV, the median TWA F_I_O_2_ of 0.8 (0.65-0.94), V_T_ of 6.1 (5.3-7.0) ml/kg, ΔP of 17 (15-20) cm H_2_O, and PEEP of 4 (4-5) cm H_2_O was applied. Incidence rate of PPCs was 25.9%, and F_I_O_2_ was independently associated with the occurrence of PPCs in multivariate logistic regression. The adjusted odds ratio per F_I_O_2_ increase of 0.1 was 1.30 (95% confidence interval: 1.04-1.65, *P* = 0.0195).

**Conclusions:**

High F_I_O_2_ was applied to the majority of patients during OLV, whereas low V_T_ and slight degree of PEEP were commonly used in our survey. Our findings suggested that a higher F_I_O_2_ during OLV could be associated with increased incidence of PPCs.

**Electronic supplementary material:**

The online version of this article (10.1186/s12871-018-0476-x) contains supplementary material, which is available to authorized users.

## Background

Postoperative pulmonary complications (PPCs) affect morbidity, mortality, length of hospital stay [1, 2] and are at least as frequent as cardiovascular complications [2]. Therefore, PPCs are one of the most serious problems during perioperative period [2, 3]. The incidence of PPCs depends on patients’ co-morbidity, surgical procedures and anesthetic factors [1, 3]. Among these, intraoperative ventilator settings are suggested to be one of the most crucial factors [4].

To prevent the occurrence of PPCs, intraoperative lung protective ventilation, mainly comprised of low tidal volume (V_T_), slight degree of positive end-expiratory pressure (PEEP), and limited airway pressure, has been reviewed [5–8]. According to several studies in open abdominal surgery, this approach improved not only postoperative respiratory function [8] but also clinical outcomes [5, 7]. This lung protective strategy has been steadily filtering into our ventilation strategy as a standard clinical practice.

In one-lung ventilation (OLV), it is indicated that high V_T_ and inspiratory airway pressure are risk factors for acute lung injury after thoracic surgery [9–11], while high ventilator support is sometimes needed during OLV to maintain patient’s oxygenation and eliminate carbon dioxide. However, the evidence for optimal ventilator settings during OLV remains insufficient. Consequently, there are numerous variations of ventilator settings, including inspired oxygen fraction (F_I_O_2_) as well as V_T_ and PEEP, due to specific pathophysiology and historical background [12–15], especially for the management of oxygen concentrations [13–16].

In this clinical study, we investigated the current practice of intraoperative ventilation during OLV in adult patients undergoing thoracic surgery. Furthermore, we tested whether the intraoperative ventilator settings were associated with the incidence of PPCs after thoracic surgery.

## Methods

### Study design, setting, and participants

A two-center prospective observational study was conducted from April 2014 to October 2014 in Japan. Participating hospitals included an academic tertiary care hospital and a community hospital. This study was approved by the institutional ethics review board (IRB) of Okayama University Hospital (No. 1922) and Fukuyama City Hospital (No. 182). The requirement for written informed consent was waived by each IRB. We screened consecutive patients over the age of 20 who were scheduled for a thoracic surgical procedure and required general anesthesia with OLV. We excluded emergency surgery, re-operative surgery, and patients who did not receive OLV. There was no specific protocol for perioperative management at the participating hospitals.

### Data source and collection

We investigated perioperative information, including preoperative characteristics, details of surgery and anesthesia, and postoperative course. Demographics and clinical data were extracted from electronic medical records. The preoperative data included sex, age, Assess Respiratory Risk in Surgical Patients in Catalonia (ARISCAT) score [17], preoperative respiratory function, and preoperative percutaneous oxygen saturation (SpO_2_). We collected anesthetic and surgical information, such as surgical procedures, types of general anesthesia, use of epidural anesthesia, and airway management as well as duration of procedure, anesthesia, and OLV. Total blood loss and volume of infusion were also collected. Minimum SpO_2_ throughout the course of anesthesia was recorded.

During OLV (0, 30, 60, and 120 min after the start of OLV and at the end of OLV), the following variables were recorded: ventilator mode, F_I_O_2_, V_T_ corrected for predicted body weight (PBW), driving pressure (ΔP) (peak inspiratory pressure minus PEEP on both pressure control and volume control ventilation), and PEEP. These data were collected by attending anesthesiologists. PBW was calculated as follows: for men, 50 + 0.91 (height (cm) - 152.4); and for women, 45.5 + 0.91 (height (cm) - 152.4) [18].

### Quantitative variables and bias

To avoid surveillance bias, time weighted average (TWA) of ventilation parameters was calculated for the first 2 h of OLV. TWA was determined by summing the mean value between consecutive time points (0, 30, 60, and 120 min after the start of OLV) multiplied by the period of time between consecutive time points and then divided by the total time. We calculated and assessed TWA of F_I_O_2_, V_T_, ΔP, and PEEP during OLV.

### Outcome measures

The primary outcome was the incidence of PPCs occurring within 7 days of thoracotomy. PPCs included pneumonia, pleural effusion, atelectasis, prolonged air leakage, pulmonary embolism and respiratory failure diagnosed according to the definitions (Table 1), which referred to previous studies [17, 19, 20]. In each center, a predetermined researcher evaluated all patients in accordance with the definitions of PPCs. To investigate the length of hospital stay (LOS) and mortality, patients were followed-up until hospital discharge or death (whichever occurred first).

**Table 1:** The definition of PPCs

PPCs	Definition
Pneumonia [19]	1. Presence of new or progressive infiltrates on chest radiograph 2. Fever (> 38 °C) or leukocyte count (< 4000, ≥12,000 WBC/mm^3^) 3. New or changed sputum, tachypnea, impaired gas exchange
Pleural effusion [17]	Chest radiograph demonstrating blunting of the costophrenic angle or loss of the sharp silhouette of the hemidiaphragm on the nonoperative side
Atelectasis [17]	Opacities evidenced on chest radiograph with a shift of the mediastinum, hilum, or hemidiaphragm toward the affected area
Prolonged air leakage [20]	Air leak requiring insertion of new chest tube or ≥7 days of postoperative chest tube drainage
Pulmonary embolism [20]	Pulmonary arteriogram or ventilation/perfusion radioisotope scan documenting thrombus
Respiratory failure [20]	Postoperative ventilator dependence ≥24 h or Need of reintubation or noninvasive ventilation

*PPCs* postoperative pulmonary complications

### Statistical analysis

Variables were assessed for normality. Categorical data were compared using chi-square tests or Fisher exact tests and reported as n (%). Continuous normally distributed variables were compared using Student *t* tests and reported as means (standard deviation), while non-normally distributed data were compared using Wilcoxon rank-sum tests and reported as medians (interquartile range). Univariate analysis was performed to compare perioperative characteristics between patients with and without PPCs. A multivariate logistic regression analysis was performed to estimate the associations between intraoperative ventilator settings and PPCs, adjusting for ARISCAT score and all univariate relevant factors that discriminate between the two groups. To explore subgroup differences in associations between the ventilator settings and PPCs, the same multivariate analyses were performed for subgroups classified according to the ARISCAT score, preoperative SpO_2_ and surgical procedures, respectively. All analyses were performed using JMP version 8.0.2 (SAS Institute, Cary, NC, USA). *P* < 0.05 was considered statistically significant. This manuscript adheres to the applicable Strengthening the Reporting of Observational Studies in Epidemiology (STROBE) guidelines.

## Results

### Participants characteristics

**Fig. 1:**
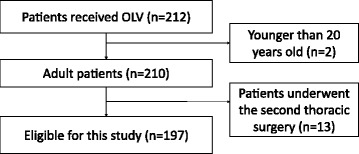
Study flow diagram. *OLV* one-lung ventilation

Overall, 212 cases underwent thoracic surgery with OLV during the study period. Two patients were younger than 20 years old, and 13 cases underwent thoracic surgeries twice during the study period. Thus, 197 patients met the eligibility criteria (Fig. 1).

Baseline characteristics and intraoperative procedures of all patients are noted in Additional file 1. Most patients (*n* = 190, 96.4%) had an intermediate or high risk of having PPCs according to the ARISCAT score. More than 80% of patients underwent lung resections; however, there was no patient who underwent pneumonectomy.

### Main results

**Fig. 2:**
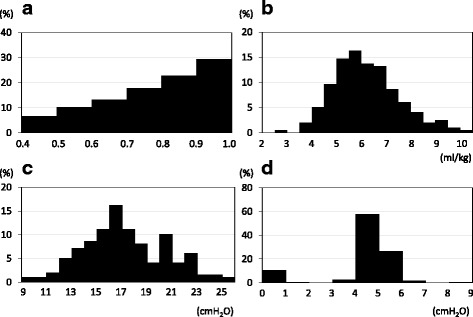
Distribution of ventilator settings during one-lung ventilation. Each graph represents the distributions of TWA values during one-lung ventilation: (**a**) F_I_O_2_, (**b**) V_T_, (**c**) ΔP, and (**d**) PEEP. *TWA* time weighted average, *F*_*I*_*O*_*2*_ inspiratory oxygen fraction, *V*_*T*_ tidal volume, *ΔP* driving pressure, *PEEP* positive end-expiratory pressure

Pressure control ventilation (PCV) was utilized in most cases (*n* = 181, 92%). At the start of OLV, median F_I_O_2_ was 1.0 (0.8-1.0). Specifically, an F_I_O_2_ of 1.0 was applied as an initial setting for more than 60% of all patients. In other initial settings, median V_T_ was 6.1 (5.2-7.3) ml/kg, and median ΔP was 16 (14-20) cm H_2_O. PEEP was applied in 171 patients (87%) at a median level of 4 (4-5) cm H_2_O. The distributions of ventilator settings throughout OLV are shown as TWA values in Fig. 2. Median TWA F_I_O_2_ was 0.8 (0.65-0.94), and 83% of patients received TWA F_I_O_2_ ≥ 0.6. Other median TWA values, such as V_T_, ΔP, and PEEP, were at almost similar levels as the initial settings (V_T_, 6.1 (5.3-7.0) ml/kg; ΔP, 17 (15-20) cm H_2_O; and PEEP, 4 (4-5) cm H_2_O). As a rescue therapy, oxygen therapy to the non-ventilated lung was adopted in only five cases.

PPCs occurred in 51 of 197 cases (25.9%). Atelectasis developed in 35 patients (17.8%), prolonged air leakage in 10 (5.1%), pneumonia in 3 (1.5%), pleural effusion in 3 (1.5%), and respiratory failure in 2 (1.0%). Two cases with respiratory failure occurred with atelectasis or pleural effusion. None of the patients were diagnosed with pulmonary embolism in this period. Only one patient died during hospital stay, and overall mortality was 0.5%. Baseline characteristics and intraoperative procedures in patients with and without PPCs were shown in Table 2. There were no significant differences in preoperative baseline characteristics, surgical procedures, and intraoperative management regarding anesthesia.

**Table 2:** Baseline characteristics and intraoperative information of patients with and without PPCs

	Patients with PPCs (*N* = 51)	Patients without PPCs (*N* = 146)	*P* value
Preoperative baseline			
Age - years	67.4 ± 12.9	63.7 ± 13.2	0.094
Sex (male) - no. (%)	33 (64.7)	88 (60.3)	0.57
ARISCAT score	50 (43-50)	50 (27-50)	0.44
Preoperative SpO_2_ - %	97 (96-98)	98 (96.25-99)	0.055
%VC - %	101 ± 18	104 ± 18	0.31
FEV1.0% - %	75 ± 13	75 ± 10	0.72
Anesthesia & Operation			
Lung resection (+) - no. (%)	45 (88.2)	123 (84.2)	0.64
TIVA - no. (%)	31 (60.8)	78 (53.8)	0.39
Epidural anesthesia - no. (%)	37 (72.6)	109 (74.7)	0.77
Oxygen therapy to the non-ventilated lung - no. (%)	3 (5.9)	2 (1.4)	0.08
Duration of anesthesia - min	285 (185-362)	263 (162-333)	0.17
Duration of operation - min	205 (118-276)	194 (102-258)	0.15
Duration of OLV - min	173 (96-240)	167 (77-224)	0.33
Total volume of infusion - ml	1660 (1250-2190)	1550 (958-2100)	0.14
Total blood loss - ml	40 (10-100)	15 (10-93)	0.37
Minimum SpO_2_ - %	94 (91-96)	95.5 (93-97)	0.0053

Baseline and procedural characteristics are shown as n (%), means ± standard deviation or medians (interquartile range)

*PPCs* postoperative pulmonary complications, *ARISCAT* Assess Respiratory Risk in Surgical Patients in Catalonia, *%VC* % vital capacity, *FEV1.0%* forced expiratory volume in one second %, *TIVA* total intravenous anesthesia, *OLV* one-lung ventilation

Among ventilator settings, only TWA F_I_O_2_ in patients with PPCs was significantly higher than that in patients without PPCs (0.85 (0.73-1.0) vs. 0.77 (0.63-0.89); *P* = 0.0032) (Table 3). There was no significant difference in TWA V_T_, TWA ΔP, and TWA PEEP between the two groups. Throughout the anesthesia, minimum SpO_2_ in patients with PPCs was significantly lower than that in patients without PPCs (94 (91-96) % vs. 95.5 (93-97) %; *P* = 0.0053). Finally, the postoperative LOS was longer in patients with PPCs (13 (8-16) days vs. 8 (7-11) days; *P* < 0.001).

**Table 3:** Ventilator setting during OLV of patients with and without PPCs

	Patients with PPCs (*N* = 51)	Patients without PPCs (*N* = 146)	*P* value
Ventilator setting during OLV			
Mode (PCV) - no. (%)	46 (90.2)	135 (92.5)	0.62
TWA F_I_O_2_	0.85 (0.73-1.0)	0.77 (0.63-0.89)	0.0032
TWA V_T_ - ml/kg	6.2 (5.2-7.4)	6.1 (5.4-7.0)	0.8495
TWA ΔP - cmH_2_O	18 (15-21)	16 (15-18)	0.0717
TWA PEEP - cmH_2_O	4 (4-5)	4 (4-5)	0.1504

Ventilator settings are shown as n (%) or medians (interquartile range)

*OLV* one-lung ventilation, *PPCs* postoperative pulmonary complications, *PCV* pressure control ventilation, *TWA* time weighted average, *F*_*I*_*O*_*2*_ inspiratory oxygen fraction, *V*_*T*_ tidal volume, *ΔP* driving pressure, *PEEP* positive end-expiratory pressure

In multivariate logistic regression model (Table 4), which was adjusted for ventilator settings (TWA F_I_O_2_, TWA ΔP, and TWA PEEP), ARISCAT score, and minimum SpO_2_, only TWA F_I_O_2_ during OLV was independently associated with the occurrence of PPCs. Odds ratio (OR) per TWA F_I_O_2_ increase of 0.1 was 1.30 (95% confidence interval (CI): 1.04-1.65, *P* = 0.0195). Other variables (TWA ΔP, TWA PEEP, ARISCAT score, and minimum SpO_2_) were not related to the occurrence of PPCs in this model.

**Table 4:** Multivariate Analysis of risk factor for PPCs

	Odds Ratio	*P* value
ARISCAT score (per 1 point)	1.02 (95% CI: 0.99-1.05)	0.3038
Minimum SpO_2_ (per 1%)	0.89 (95% CI: 0.79-1.00)	0.0544
TWA F_I_O_2_ (per 0.1)	1.30 (95% CI: 1.04-1.65)	0.0195
TWA ΔP (per 1 cmH_2_O)	1.03 (95% CI: 0.91-1.16)	0.6436
TWA PEEP (per 1 cmH_2_O)	1.09 (95% CI: 0.86-1.40)	0.4994

*PPCs* postoperative pulmonary complications, *ARISCAT* Assess Respiratory Risk in Surgical Patients in Catalonia, *CI* confidence interval, *TWA* time weighted average, *F*_*I*_*O*_*2*_ inspiratory oxygen fraction, *ΔP* driving pressure, *PEEP* positive end-expiratory pressure

### Subgroup analyses

There were significant associations between F_I_O_2_ and PPCs in patients with low or intermediate risk of having PPCs according to the ARISCAT score (OR, 1.48; 95% CI, 1.00-2.40; *P* = 0.0496), or undergoing lung resection (OR, 1.31; 95% CI, 1.03-1.70; *P* = 0.0278) (Additional file 2). Other subgroups including patients with high risk for PPCs and high or low preoperative SpO_2_, also indicated that higher F_I_O_2_ tended to be associated with higher incidence of PPCs.

## Discussion

### Key results

We conducted a prospective observational study to investigate the current practice of intraoperative ventilation and to evaluate the associations between ventilator settings during OLV and PPCs in patients undergoing thoracic surgery. We found that F_I_O_2_ of ≥0.8, V_T_ of approximately 6 ml/kg, and PEEP of approximately 4 cm H_2_O were common. Patients with PPCs received higher F_I_O_2_ during OLV, while they had lower minimum SpO_2_ than those without PPCs. However, in multivariate logistic regression analysis adjusting for ventilator settings, ARISCAT score, and minimum SpO_2_, only TWA F_I_O_2_ was associated with the occurrence of PPCs, and the adjusted OR per F_I_O_2_ increase of 0.1 was 1.30. Therefore, an increase in oxygen concentration of 10% was associated with approximately 30% increase in the risk of PPCs.

### Interpretation

We found that V_T_ was around 6 ml/kg, and PEEP was set around 4 cm H_2_O in most patients. These findings were consistent with recent studies or textbook oriented lung protective strategy [15, 21, 22]. We also found that high F_I_O_2_ was frequently used during OLV. These findings, however, were inconsistent with recent recommended management [22]. An F_I_O_2_ of 1.0 was classically a routine component of OLV [15, 23]. However, the incidence of hypoxemia during OLV has been decreasing [15, 22], and the harmful effects of high F_I_O_2_, including absorption atelectasis [24–27], production of reactive oxygen species, and increased lung injury [28, 29], have been reported. Therefore, this classic practice has been questioned and avoidance of excessive F_I_O_2_ has been proposed [15]. The latest textbook suggests that F_I_O_2_ should be titrated to maintain a stable saturation level above 92-94% during OLV [22]. However, some reports revealed that relatively high F_I_O_2_ was still applied as a common practice during both two-lung ventilation [30, 31] and OLV [13–16]. In our survey, intraoperative minimum SpO_2_ was ≥95% in 111 patients (56%), with 83% of them receiving TWA F_I_O_2_ of ≥0.6 (Additional file 3). These findings indicated that almost half of the patients may have received excessive oxygen regardless of their SpO_2_. There was low compliance with recommended standards to maintain a SpO_2_ above 92-94% during OLV.

According to our results, high F_I_O_2_ during OLV was independently associated with the increasing incidence of PPCs, and patients with PPCs had a longer LOS in the hospital. Worse clinical outcomes due to high F_I_O_2_ were previously reported in critically ill adults, including patients with chronic obstructive pulmonary disease, myocardial infarction, cardiac arrest, stroke, and traumatic brain injury [32–35]. Given the above concern, a conservative oxygenation strategy has been shown to be feasible, safe, and effective for mechanically ventilated patients in recent decades [36, 37]. Notably, conservative oxygen therapy could be associated with decreased evidence of atelectasis as well as earlier weaning from mandatory ventilation in the ICU [38]. Additionally, a recent randomized control trial of conservative oxygen therapy in ICU showed lower mortality [39].

Only a few studies investigated the effect of intraoperative F_I_O_2_ on clinical outcomes in thoracic surgery with OLV. Yang et al. reported a lower incidence of postoperative lung dysfunction and satisfactory gas exchange was provided by the lung protective strategy using F_I_O_2_ of 0.5 compared to the conventional strategy using F_I_O_2_ of 1.0 during OLV [40]. However, F_I_O_2_ was one of components in this lung protective strategy, because V_T_, PEEP, and mode of mechanical ventilation were also different between the groups. Thus, it remains uncertain whether a conservative approach to oxygen therapy during OLV is beneficial or not. To our knowledge, this is the first study to demonstrate an association between high F_I_O_2_ during OLV and the occurrence of PPCs. To confirm and dissect these findings, additional studies should be performed in different settings. Moreover, our findings support the need for randomized control trials to evaluate the safety and feasibility of conservative oxygen therapy during OLV.

### Limitations

There were several limitations in this study. First, because this was an observational study, causality was not determined. It should be noted that higher F_I_O_2_ might be confounded by the incidence of hypoxemia, which could cause PPCs. Thus, the role of F_I_O_2_ is difficult to differentiate between “unnecessary use” and “need for higher support.” However, after adjusting by ARISCAT score, minimum SpO_2_, ΔP, and PEEP to reduce potential confounding, only higher F_I_O_2_ remained statistically significant as an independent risk factor for PPCs. In subgroup analyses, F_I_O_2_ has been associated with the incidence of PPCs even in patients with comparatively lower risk for PPCs. Additionally, the present study indicated that patients might receive excessive oxygen during OLV. Therefore, we believe that intraoperative F_I_O_2_ could be titrated safely even during OLV.

Second, the incidence of PPCs could have heavily depended on our definition. There are various definitions of PPCs. For instance, pneumonia was diagnosed based on radiologic images, symptoms, laboratory findings, or antimicrobial treatment used. The diagnosis of atelectasis was based on images or bronchoscopy. In our study, we used definitions of PPCs from previous studies [17, 20] and CDC guidelines [19] as shown in Fig. 1. As a result, the incidence of PPCs in our study (25.9%) was similar to that of previous works [17, 20].

## Conclusions

In conclusion, liberal oxygen therapy as well as lung protective ventilation comprising low V_T_ and slight PEEP were common for patients undergoing thoracic surgery with OLV in our cohort. Our findings indicated that high F_I_O_2_ during OLV was associated with an increased incidence of PPCs, which is related to prolonged LOS in the hospital. These results suggested that current practices of oxygen therapy during OLV may be suboptimal and warrant further investigation.

## Additional files

Additional file 1: Baseline characteristics and intraoperative procedures of all patients. (DOCX 21 kb)

Additional file 2: Adjusted odds ratio of TWA F_I_O_2_ during OLV for the incidence of PPCs in subgroup analyses. (PPTX 79 kb)

Additional file 3: The correlation between TWA F_I_O_2_ and minimum SpO_2_. (PPTX 84 kb)

## Abbreviations

%VC: % vital capacity
ARISCAT: Assess Respiratory Risk in Surgical Patients in Catalonia
CI: Confidence interval
FEV1.0%: Forced expiratory volume in one second %
F_I_O_2_: Inspired oxygen fraction
IRB: Institutional ethics review board
LOS: Length of hospital stay
OLV: One-lung ventilation
OR: Odds ratio
PBW: Predicted body weight
PCV: Pressure control ventilation
PEEP: Positive end-expiratory pressure
PPCs: Postoperative pulmonary complications
SpO_2_: Percutaneous oxygen saturation
TIVA: Total intravenous anesthesia
TWA: Time weighted average
V_T_: Tidal volume
ΔP: Driving pressure
