# Supplementary material for: Associations between intraoperative ventilator settings during one-lung ventilation and postoperative pulmonary complications: a prospective observational study
Source: BMC Anesthesiol. 2018 Jan 25;18:13. doi: 10.1186/s12871-018-0476-x (PMC5785851; doi:10.1186/s12871-018-0476-x)
Supplement: Supplementary file 1 — Baseline characteristics and intraoperative procedures of all patients. (DOCX 21 kb) [file 12871_2018_476_MOESM1_ESM.docx]

Additional file 1 Baseline characteristics and intraoperative procedures of all patients

| Clinical variables | All patients (N = 197) |
| --- | --- |
| Age - years | 64.7 ± 13.2 |
| Sex (male) - no. (%) | 121 (61.4) |
| ARISCAT score - no. (%)  Low (<26)  Intermediate (26-44)  High (>44) | 7 (3.6)  72 (36.5)  118 (59.9) |
| Preoperative SpO_2_ - % | 98 (96-99) |
| Surgical procedure - no. (%)  Lobectomy  Segmentectomy  Wedge resection  Pleura and chest wall surgery  Mediastinal tumor | 93 (47.2)  48 (24.4)  25 (12.7)  16 (8.1)  15 (7.6) |
| Type of general anesthesia - no. (%)  Total intravenous anesthesia  Volatile anesthesia | 110 (55.8)  87 (44.2) |
| Postoperative analgesia - no. (%)  Thoracic epidural analgesia  PCA of opioid  Intercostal block | 146 (74.1)  33 (16.8)  30 (15.2) |
| Lung separation techniques - no. (%)  Double-lumen endotracheal tube  Endobronchial blocker | 185 (93.9)  12 (6.1) |
| Duration - min  Anesthesia  Operation  One-lung ventilation | 264 (171-339)  194 (110-261)  225 (87-168) |
| Total volume of infusion - ml  Total blood loss - ml | 1600 (1075-2100)  20 (10-100) |

Baseline and procedural characteristics are shown as n (%), means ± standard deviation or medians (interquartile range)

*ARISCAT* Assess Respiratory Risk in Surgical Patients in Catalonia, *PCA* patient-controlled analgesia
