# Supplementary material for: Associations between intraoperative ventilator settings during one-lung ventilation and postoperative pulmonary complications: a prospective observational study
Source: BMC Anesthesiol. 2018 Jan 25;18:13. doi: 10.1186/s12871-018-0476-x (PMC5785851; doi:10.1186/s12871-018-0476-x)
Supplement: Supplementary file 2 — Adjusted odds ratio of TWA FIO2 during OLV for the incidence of PPCs in subgroup analyses. (PPTX 79 kb) [file 12871_2018_476_MOESM2_ESM.pptx]

## Slide 1
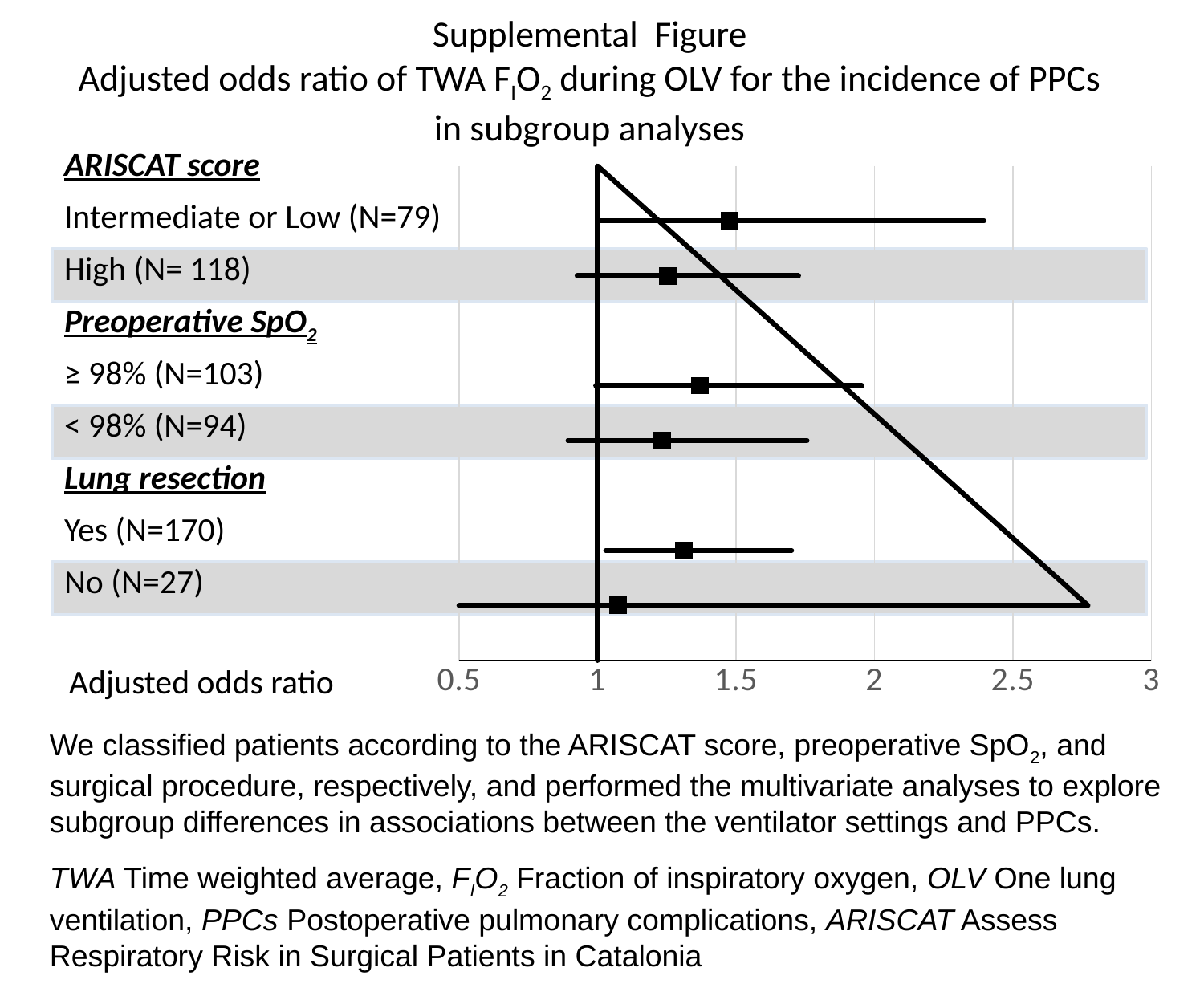

# Supplemental Figure Adjusted odds ratio of TWA FIO2 during OLV for the incidence of PPCs in subgroup analyses
| ARISCAT score |
| --- |
| Intermediate or Low (N=79) |
| High (N= 118) |
| Preoperative SpO2 |
| ≥ 98% (N=103) |
| < 98% (N=94) |
| Lung resection |
| Yes (N=170) |
| No (N=27) |
### Chart
| Category | | |
|---|---|---|
Adjusted odds ratio
We classified patients according to the ARISCAT score, preoperative SpO2, and surgical procedure, respectively, and performed the multivariate analyses to explore subgroup differences in associations between the ventilator settings and PPCs.
TWA Time weighted average, FIO2 Fraction of inspiratory oxygen, OLV One lung ventilation, PPCs Postoperative pulmonary complications, ARISCAT Assess Respiratory Risk in Surgical Patients in Catalonia
