# Supplementary material for: Associations between intraoperative ventilator settings during one-lung ventilation and postoperative pulmonary complications: a prospective observational study
Source: BMC Anesthesiol. 2018 Jan 25;18:13. doi: 10.1186/s12871-018-0476-x (PMC5785851; doi:10.1186/s12871-018-0476-x)
Supplement: Supplementary file 3 — The correlation between TWA FIO2 and minimum SpO2. (PPTX 84 kb) [file 12871_2018_476_MOESM3_ESM.pptx]

## Slide 1
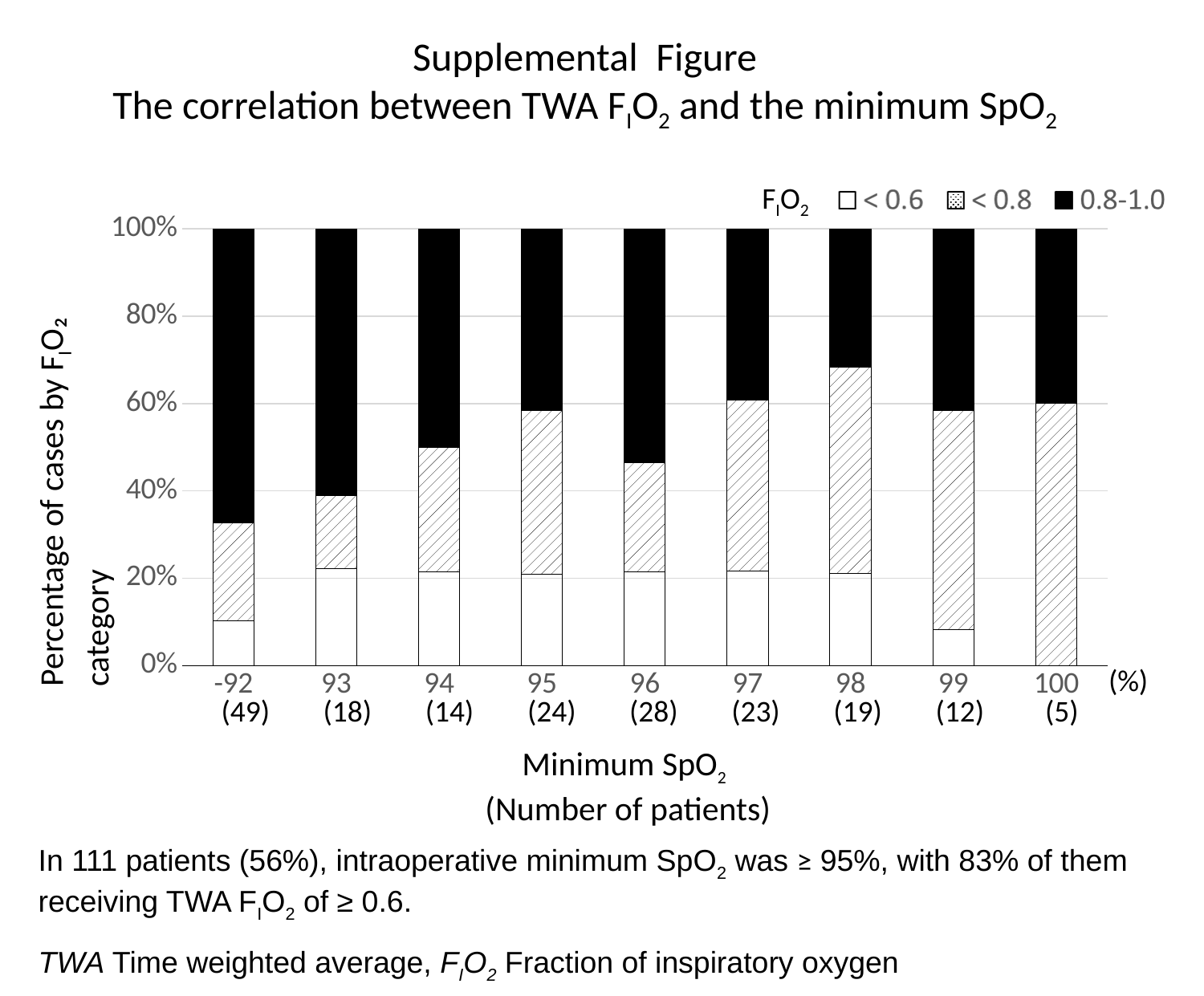

# Supplemental Figure The correlation between TWA FIO2 and the minimum SpO2
FIO2
Percentage of cases by FIO₂ category
### Chart
| Category | < 0.6 | < 0.8 | 0.8-1.0 |
|---|---|---|---|
| -92 | 5.0 | 11.0 | 33.0 |
| 93 | 4.0 | 3.0 | 11.0 |
| 94 | 3.0 | 4.0 | 7.0 |
| 95 | 5.0 | 9.0 | 10.0 |
| 96 | 6.0 | 7.0 | 15.0 |
| 97 | 5.0 | 9.0 | 9.0 |
| 98 | 4.0 | 9.0 | 6.0 |
| 99 | 1.0 | 6.0 | 5.0 |
| 100 | None | 3.0 | 2.0 |(%)
| (49) | (18) | (14) | (24) | (28) | (23) | (19) | (12) | (5) |
| --- | --- | --- | --- | --- | --- | --- | --- | --- |
Minimum SpO2
(Number of patients)
In 111 patients (56%), intraoperative minimum SpO2 was ≥ 95%, with 83% of them receiving TWA FIO2 of ≥ 0.6.
TWA Time weighted average, FIO2 Fraction of inspiratory oxygen
